# Morphological and Transcriptomic Analysis of the Inhibitory Effects of *Lactobacillus plantarum* on *Aspergillus flavus* Growth and Aflatoxin Production

**DOI:** 10.3390/toxins11110636

**Published:** 2019-11-01

**Authors:** Yueju Zhao, Chenxi Zhang, Yawa Minnie Elodie Folly, Jinghua Chang, Yan Wang, Lu Zhou, Heping Zhang, Yang Liu

**Affiliations:** 1 Key Laboratory of Agro-products Quality and Safety Control in Storage and Transport Process, Ministry of Agriculture and Rural Affairs/Institute of Food Science and Technology, Chinese Academy of Agricultural Sciences, Beijing 100193, China; zhaoyueju@caas.cn (Y.Z.); zhangchenxi@caas.cn (C.Z.); yelodieminnie@yahoo.fr (Y.M.E.F.); wangyan@caas.cn (Y.W.); 2 College of Science, Liaoning Technical University, Fuxin 123000, China; cjh2975@sina.com; 3 Biological Testing and Analysis Department, Guangdong Provincial Institute of Food Inspection, Guangzhou 51000, China; zhoulu@caas.cn; 4 Key Laboratory of Dairy Biotechnology and Engineering, Ministry of Education, Key Laboratory of Dairy Products Processing, Ministry of Agriculture, Inner Mongolia Agricultural University, Hohhot 010018, China

**Keywords:** *Aspergillus flavus*, *Lactobacillus plantarum*, inhibition, transcriptomics, bioinformatics, SEM, RNA-seq

## Abstract

*Lactobacillus plantarum*, as a natural bio-preservative, has attracted a great deal of attention in recent years. In this study, 22 *L. plantarum* strains were tested against the aflatoxin-producing fungus, *Aspergillus flavus*; strain IAMU80070 showed the highest antifungal activity. At a concentration of 5 × 10^5^ colony-forming units (CFU) mL^−1^, it completely inhibited *A. flavus* growth and decreased aflatoxin production by 93%. Furthermore, ultrastructural examination showed that IAMU80070 destroyed the cellular structure of hyphae and spores. To explore the inhibitory effect of IAMU80070 on *A. flavus* at the transcriptional level, transcriptome data were obtained and subjected to Gene Ontology (GO) and Kyoto Encyclopedia of Genes and Genomes (KEGG) analyses. The aflatoxin biosynthetic process was the most significantly downregulated functional category, while genes implicated in the synthesis and organization of cell wall polysaccharides were upregulated. Quantitative real-time PCR results verified the credibility and reliability of the RNA sequencing data. These results provided insight into the transcriptome of *A. flavus* in response to the antagonistic effects of *L. plantarum* IAMU80070.

## 1. Introduction

*Aspergillus flavus*, a soil-borne, saprophytic fungus, infects fatty acid-rich food and animal feed [1]. Upon infection, the fungus produces aflatoxins such as aflatoxin B_1_ (AFB_1_), AFB_2_, AFG_1_, and AFG_2_ [2] that are highly carcinogenic and mutagenic, and are immunosuppressive secondary metabolites [3,4]. Contamination by *A. flavus* poses a serious threat to food safety. Biocontrol of *A. flavus* with *lactic acid bacteria* (LAB) is considered an eco-friendly approach [5,6].

LAB hold great potential for use as natural food preservatives due to their long safe history of use in fermentation and the production of antifungal compounds [6]. *Lactobacillus plantarum* is one of the most popular LAB species groups employed in food production. *L. plantarum* strains from different food matrices were screened for their antifungal activity against *A. flavus* and other common contaminant molds responsible for the spoilage of cereals; the strongest antifungal strain displayed the best biopreservative effects [7]. *L. plantarum* isolated from fermented olives could inhibit the growth of *A. flavus* and detoxify aflatoxin B_1_ on olives [8]. *L. plantarum* strains from fermented cereal Kunu [9] or from Kenyan traditional fermented milk and maize products [10] could completely inhibit the growth of *A. flavus*. *L. plantarum*, together with other lactobacilli strains, improved the quality of maize grain silage by inhibiting the growth of pathogens and decreasing mycotoxins [11]. To explore how *L. plantarum* inhibits the growth of *A. flavus*, various antifungal metabolites produced by *L. plantarum* have been characterized, including 3-phenyllactic acid (PLA) [12,13,14,15], hydroxyphenyllactic acid [7,15,16], indole lactic acid [15], delta-dodecalactone [17], and cyclic dipeptides [18,19]. These chemicals can destroy the structure of the cell membrane, disrupt intracellular pH homeostasis, and inhibit essential metabolic reactions, resulting in growth inhibition of *A. flavus* [20].

The cell-free supernatant (CFS) of *L. plantarum* was reported to play a major role in the inhibition of aflatoxin production [15]. However, morphological and transcriptomic studies on *A. flavus* co- cultivated with *L. plantarum* have not been reported. These studies would aid the development of the biological control of *A. flavus* and aflatoxin contamination using *L. plantarum*. Herein, *L. plantarum* strains isolated from fermented food and milk products were screened for *A. flavus* growth inhibition. Strain IAMU80070 showed the highest antifungal activity, and was further investigated to (1) evaluate the inhibitory effects of *L. plantarum* IAMU80070 on *A. flavus* and aflatoxin production; (2) examine the ultrastructural changes occurring in hypha cells of *A. flavus* during interaction with *L. plantarum* IAMU80070 by scanning electron microscopy (SEM); and (3) analyze transcriptomic changes in *A. flavus* to investigate the putative biocontrol mechanism at the transcriptional level.

## 2. Results

### 2.1. Screening of L. plantarum against A. flavus

**Figure 1:**
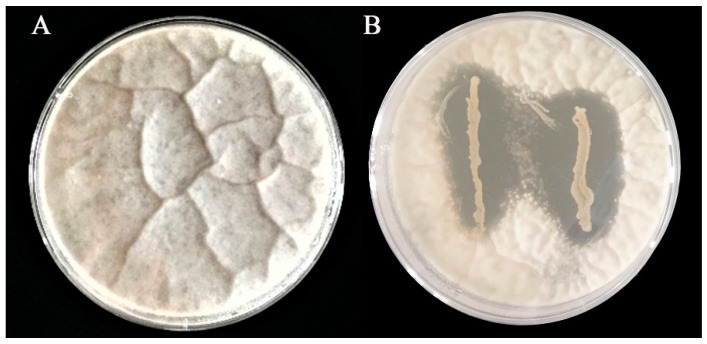
In vitro, antagonistic effect of *L*. *plantarum* on *A. flavus* on double-layer plates after 5 days’ incubation at 28 °C. (**A**) *A*. *flavus* without *L*. *plantarum* IAMU80070. (**B**) *A. flavus* with *L*. *plantarum* IAMU80070 streaked on MRS plates.

Twenty-two bacterial strains isolated from dairy products, kimchi, sour porridge, and sour dough were screened for their potential to inhibit the growth of *A. flavus*. These strains showed a wide range of inhibitory effects on *A. flavus* growth after a 5-day incubation at 28 °C (Table 1). Strain IAMU80070 displayed the highest apparent inhibitory activity, and was therefore selected for further characterization and investigation (Figure 1).

### 2.2. Inhibitory Effect of L. plantarum IAMU80070 on A. flavus Growth

**Figure 2:**
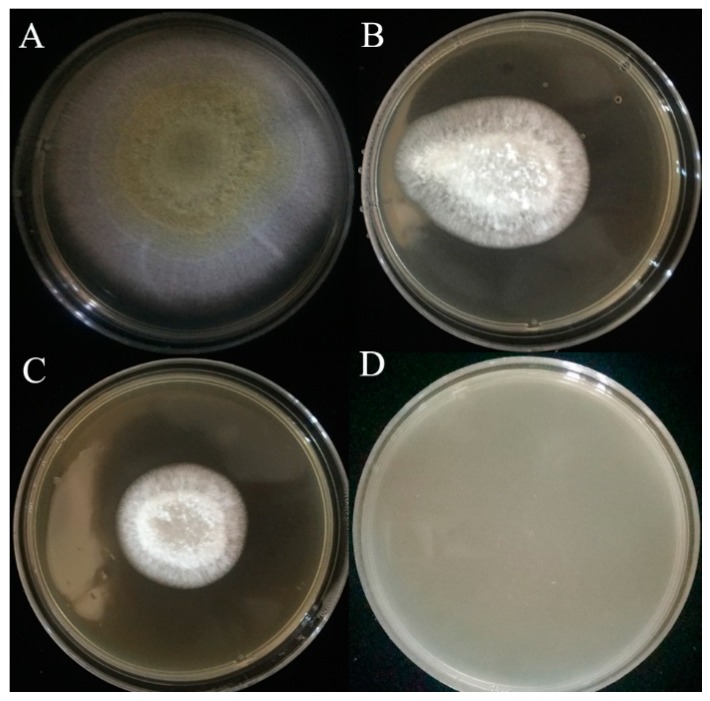
Effect of different concentrations of *L. plantarum* IAMU80070 on the growth of *A. flavus*. (**A**) Without *L. plantarum* IAMU80070. (**B**) *L. plantarum* IAMU80070 at 5 × 10^4^ CFU/mL. (**C**) *L. plantarum* IAMU80070 at 1.5 × 10^5^ CFU/mL. (**D**) *L. plantarum* IAMU80070 at 5 × 10^5^ CFU/mL.

The growth of *A. flavus* was analyzed in the presence of different concentrations of IAMU80070. As shown in Figure 2, the mycelium diameter of *A. flavus* was significantly decreased with increasing IAMU80070 concentration on MRS-PDA double-layer plates. When the concentration of IAMU80070 reached 5 × 10^5^ CFU/mL, growth of *A. flavus* was completely inhibited.

### 2.3. Inhibitory Effect of L. plantarum IAMU80070 on Aflatoxin Production

**Figure 3:**
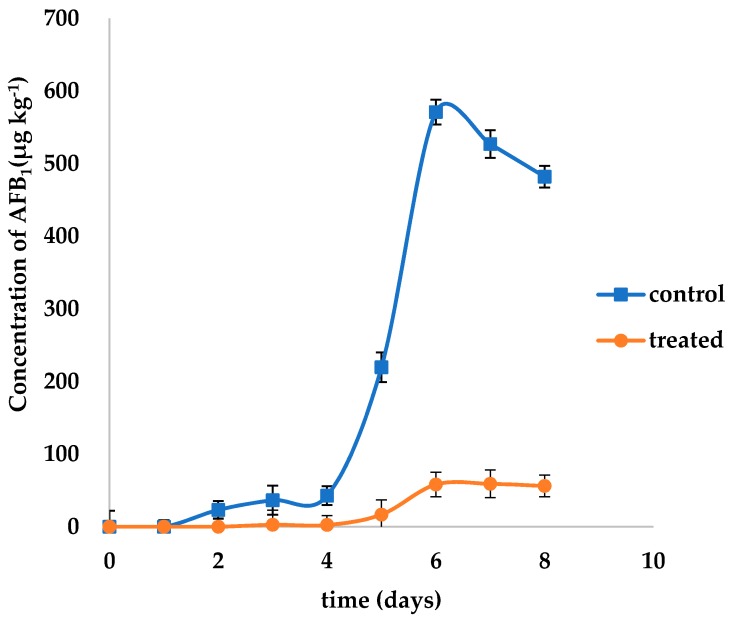
Inhibitory effect of *L. plantarum* IAMU80070 on AFB_1_ production by *A. flavus*.

When co-cultured with *L. plantarum* IAMU80070 at a concentration of 1.5 × 10^5^ CFU/mL, aflatoxin production of *A. flavus* was greatly inhibited (Figure 3). The highest concentration of aflatoxin in agar was only 59.1 μg/kg after incubation at 28 °C for 6 days, while 559.8 μg/kg aflatoxin was observed in the control lacking *L. plantarum* IAMU80070.

### 2.4. Effects of L. plantarum IAMU80070 on the Degradation and/or Sequestration of Aflatoxins

An aflatoxins reduction assay was carried out in order to detect whether *L. plantarum* IAMU80070 had the function of degrading and/or sequestrating aflatoxins at the same time as inhibiting the synthesis of aflatoxins. Unfortunately, it was found that *L. plantarum* IAMU80070 was not able to remove AFB_1_, AFB_2_, AFG_1_, and AFG_2_ effectively (Table 2). *L. plantarum* IAMU80070 seemed unable to reduce the numbers of aflatoxins which already existed.

### 2.5. Effects of L. plantarum IAMU80070 on the Ultrastructure of A. flavus.

**Figure 4:**
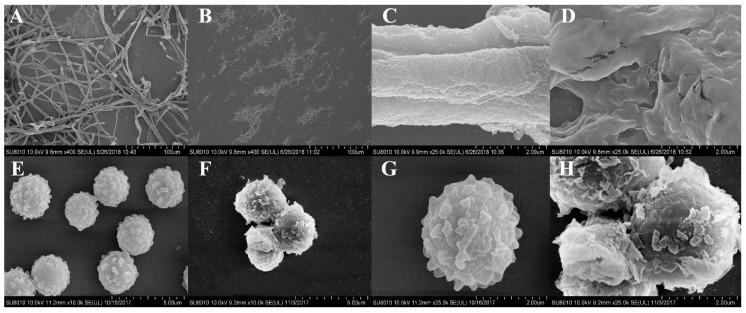
SEM analysis of the antagonistic effects of *L. plantarum* IAMU80070 against *A. flavus*. (**A**,**C**) Hyphae of *A. flavus*. (**B**,**D**) Hyphae of *A. flavus* co-cultured with *L. plantarum* IAMU80070. (**E**,**G**) Spores of *A. flavus*. (**F**,**H**) Spores of *A. flavus* co-cultured with *L. plantarum* IAMU80070.

SEM analysis revealed apparently healthy hyphae which were regular in shape and with a compact structure when *A. flavus* was cultured without the *L. plantarum* strain (Figure 4A,C). Noticeable morphological changes in hyphae were observed when cocultured with IAMU80070. Most strikingly, the mycelia of *A. flavus* were degraded and broken into small pieces (Figure 4B), and the mycelia has becomed blurred (Figure 4D). Additionally, marked hyphal surface flaking was observed on the spores (Figure 4F,H) which were different with ones without *L. plantarum* strain (Figure 4E,G). Strain IAMU80070 induced stripping of the spore surface, leading to debris accumulation or dispersion.

### 2.6. Chitinase Activity of L. plantarum IAMU80070

**Figure 5:**
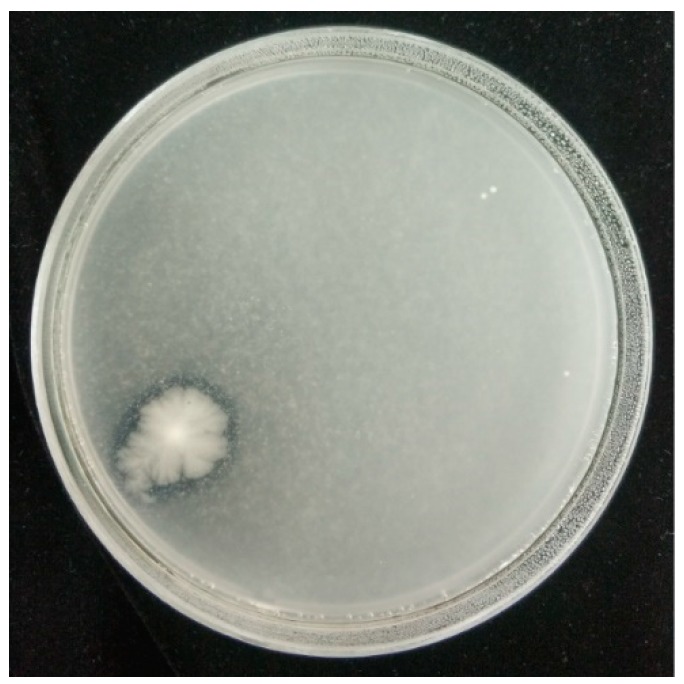
Chitinase activity of L. plantarum IAMU80070.

Further, chitinase activity of IAMU80070 was detected on chitin-amended media, and after incubation at 28 °C for 7 days, a clear halo around the colony was observed (Figure 5), indicating that *L. plantarum* IAMU80070 had secreted chitinase.

### 2.7. Comparison of Gene Expression among RNA-seq Groups

**Figure 6:**
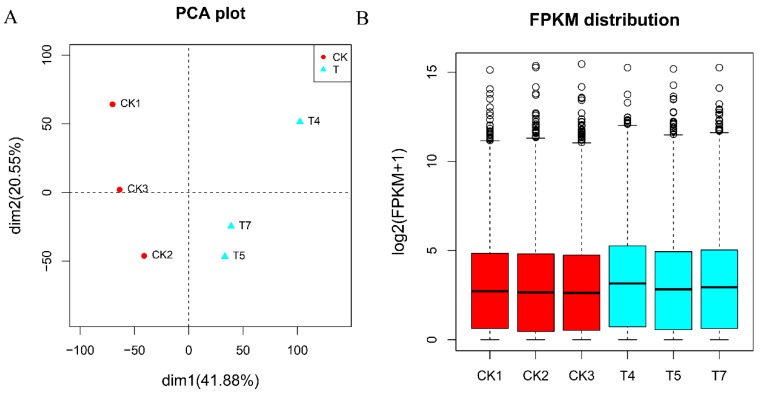
Overall expression levels in the two groups. (**A**) PCA plot of the two groups. (**B**) Boxplot of overall expression levels in the two groups.

*A. flavus* grown in the presence of *L. plantarum* IAMU80070 was set as group T, while that grown without *L. plantarum* IAMU80070 was set as the control, named group CK. An average of 40 million reads were observed for each RNA-seq library, and the mapping rate ranged from 85–88% (Table 3). The sample homogeneity of the sequenced samples was analyzed, and results were shown to be highly reproducible and reliable (Figure 6A). The expression levels of six RNA-seq libraries were represented by boxplot profiles (Figure 6B). The gene expression levels in all three samples were highly similar, indicating that RNA-seq data were reliable.

When *A. flavus* was grown with *L. plantarum* IAMU80070 (group T), 223 genes were identified as differentially expressed genes ((DEGs) (log2|fold-change| > 2, *p*-value ≤ 0.05). Among these, 111 were upregulated and 112 were downregulated (Supplemental Table S1).

### 2.8. Functional Analysis and Classification of DEGs

**Figure 7:**
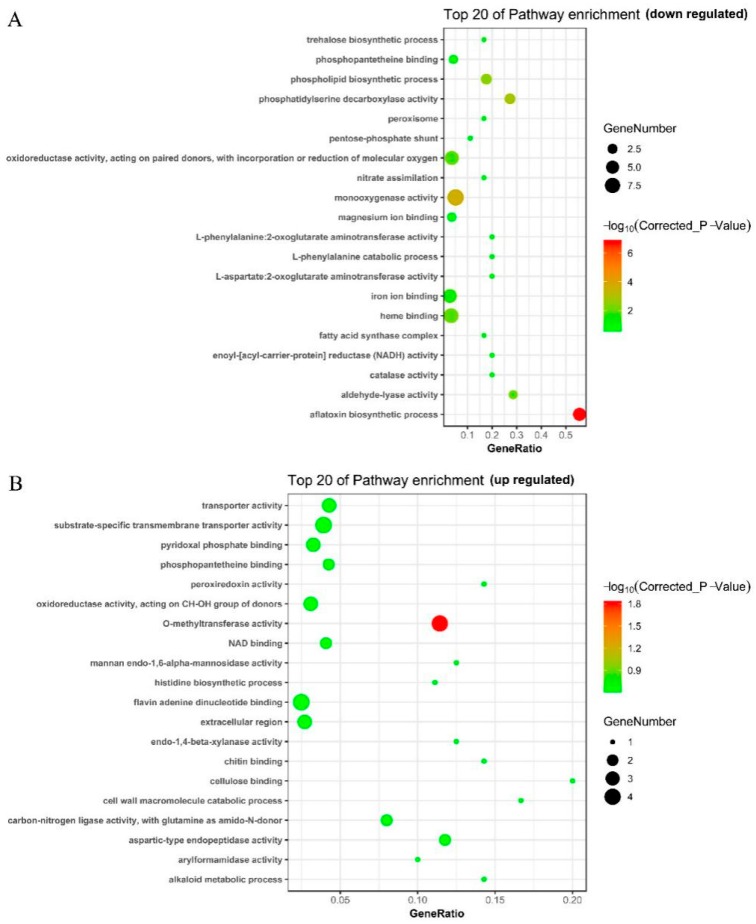
Top 20 enriched pathways based on GO analysis. (**A**) Top 20 downregulated pathways. (**B**) Top 20 upregulated pathways.

Further GO analysis was performed to analyze the functional classifications of DEGs and identify the top 20 associated pathways. Compared with the control (CK) group (Figure 7A), genes involved in important metabolic activities, including the pentose-phosphate shunt, trehalose biosynthetic process, phospholipid biosynthetic process, phosphatidylserine decarboxylase activity, nitrate assimilation, L-phenylalanine catabolic process, aminotransferase activity, and aflatoxin biosynthetic process, were downregulated in group T. Additionally, genes implicated in regulating redox status, such as peroxisomes, oxidoreductase activity, monooxygenase activity, enoyl-(acyl-carrier protein) reductase (NADH) activity, and catalase activity, were also downregulated to varying degrees in group T. Based on the p-value, number of genes, and enrichment factor, the aflatoxin biosynthetic process was the most significantly downregulated functional category in group T.

Regarding upregulated genes (Figure 7B), genes implicated in the synthesis and organization of cell wall polysaccharides, such as mannan endo-1,6-alpha-mannosidase activity, endo-1,4-beta-xylanase activity, chitin binding, cellulose binding, and cell wall macromolecule catabolic process were upregulated. O-methyltransferase activity was significantly upregulated (*p*-value ≤ 0.01, corrected *p*-value ≤ 0.05).

The KEGG pathway database was explored to identify the biological pathways associated with DEGs; only four KEGG pathways (aflatoxin biosynthesis, pentose phosphate pathway, pyruvate metabolism, and carbon metabolism) were identified (*p*-value ≤ 0.05, corrected *p*-value ≤ 0.05; Table 4).

### 2.9. Analysis of DEGs Associated with Aflatoxin Synthesis

**Figure 8:**
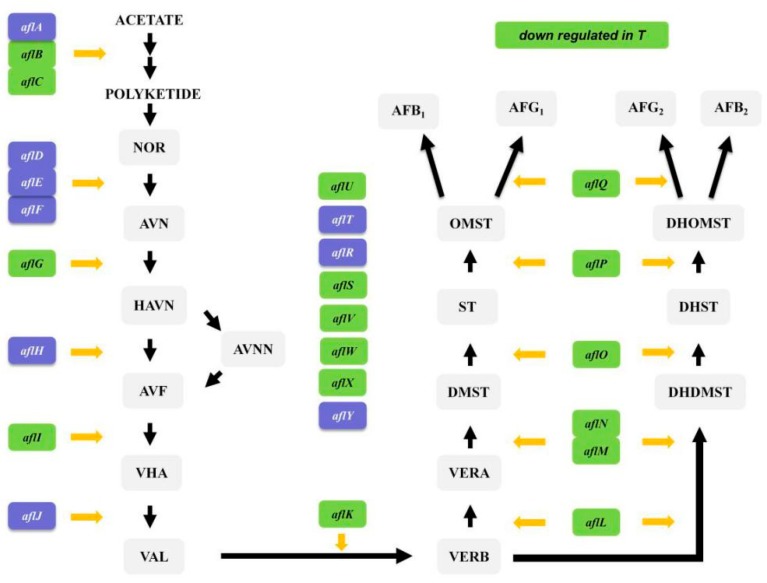
DEGs of aflatoxin synthetic pathways determined by KEGG enrichment analysis. Genes colored green are downregulated when *A. flavus* is grown in the presence of *L. plantarum* IAMU80070 (group T) compared with controls (group CK).

Based on the results of both GO and KEGG analyses, aflatoxin synthesis genes were obviously downregulated. Therefore, DEGs related to aflatoxin synthesis were further analyzed. In total, 16 genes in the aflatoxin synthesis pathway were downregulated, and most genes involved in synthesizing the aflatoxin skeleton were downregulated. In particular, genes downstream in the synthesis pathway (from *aflK* to *aflQ*) were all downregulated (Figure 8).

### 2.10. qRT-PCR Validation of RNA-seq Data

**Figure 9:**
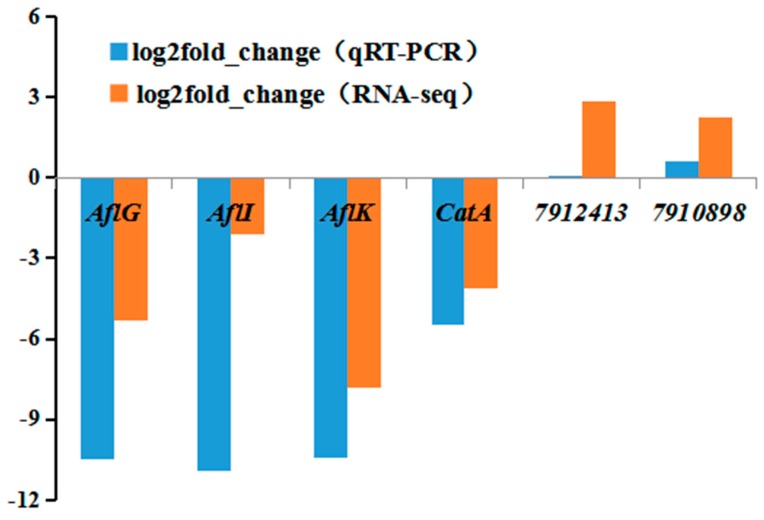
qRT-PCR analysis of selected DEGs for validation of RNA-seq data.

The qRT-PCR was used to validate the RNA-seq data. Six genes were chosen out of the 223 identified DEGs. Based on GO and KEGG analyses, three types of genes (implicated in aflatoxin synthesis, regulating redox status, and synthesis of the cell wall) were selected. Specifically, three out of the 16 aflatoxin synthesis pathway genes were randomly selected, along with *aflG*, *aflI*, and aflK. The *CatA* gene required for the response to oxidative stress was chosen, along with *Cel413* (Entrez gene ID: 7912413) and *chi100* (Entrez gene ID: 7910898), both encoding polysaccharide-related proteins. The results of qRT-PCR were in good agreement with the RNA-seq data (Figure 9).

### 2.11. Antifungal Activity in Bread

**Figure 10:**
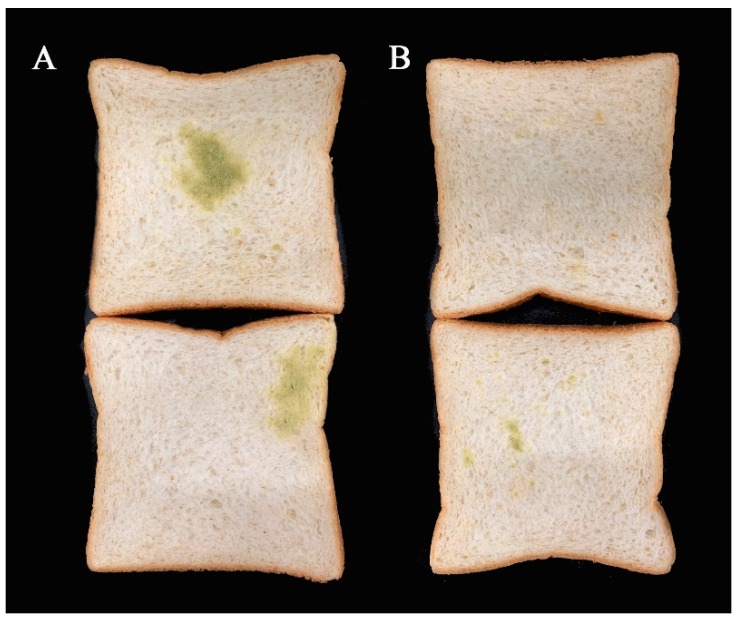
Antifungal activity of IAMU80070 in bread. (**A**) *A. flavus* and MRS medium. (**B**) *A. flavus and* IAMU80070 suspension.

After 3 days of culture at 28 °C, mildew was observed on the bread (Figure 10). It was found that only the control had serious *A. flavus* contamination, while the *L. plantarum*-treated sample had a little. This indicated that IAMU80070 has an anti-mildew effect on bread.

### 2.12. Antifungal Activity in Peanut Meal

**Figure 11:**
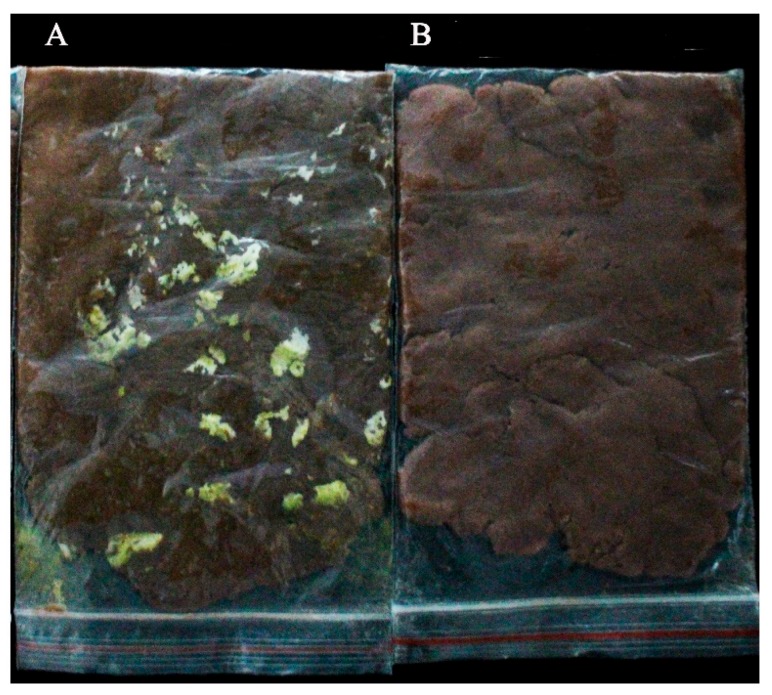
Antifungal activity of IAMU80070 in peanut meal. (**A**) *A. flavus* and MRS medium. (**B**) *A. flavus and* IAMU80070 suspension.

After 3 days of culture at 28 °C, mildew was observed on the peanut meal (Figure 11). It was found that only the control had serious *A. flavus* contamination, while the *L. plantarum*-treated sample had a little. This indicated that IAMU80070 has an anti-mildew effect on peanut meal.

## 3. Discussion

*A. flavus* can infect and contaminate preharvest and postharvest seed crops with highly toxic aflatoxins. *L. plantarum* strains from different food sources have been reported for their ability to control *A. flavus* and aflatoxins. *L. plantarum* strains possess the ability to reduce the toxicity of aflatoxins through binding activities or promoting gut microbial homeostasis in broiler chickens exposed to aflatoxin B_1_ [21,22,23]. In our current study, strain IAMU80070 was isolated from kimchi and characterized. This strain could effectively inhibit *A. flavus* growth and aflatoxin B_1_ production, and had an anti-mildew effect on bread and peanut meal.

However, it could not degrade or bind aflatoxins including B_1_, B_2_, G_1_, and G_2_. Further screening of *L. plantarum* strains with the capacity for inhibition of fungal growth, aflatoxin synthesis, and aflatoxin detoxification or binding is underway in our laboratory.

Fungal cell walls are essential for cell morphogenesis. At the morphological level, we found obvious changes in the surfaces of hyphae and spores. Similar results were obtained in our previous research on the antagonistic effects of *Bacillus subtilis* SG6 against *F. graminearum* D187 [24]. SG6 induces changes in the hyphae surface by producing chitinases that degrade chitin, an essential component of the fungal cell wall that plays an important role under cell wall stress conditions. In this study, chitinase activity of IAMU80070 was also detected on chitin-amended media. We observed clearance halos around and beneath the growth of IAMU80070, indicating that chitinase production is also involved in the biocontrol of *A. flavus* by strain IAMU80070.

The composition of fungal cell walls influences fungal ecology and the highly-regulated responses to environmental conditions and imposed stresses [25]. At the transcriptional level, the five genes implicated in the synthesis and organization of cell wall polysaccharides were upregulated based on GO analysis (Figure 7). This indicated that *A. flavus* attempted to fight back and struggled to survive by adjusting its cell wall composition when faced with stress caused by IAMU80070.

Aflatoxins are highly oxygenated polyketide secondary metabolites; their synthesis is triggered and intensified by the build-up of reactive oxygen species. Knowledge concerning the inhibition of aflatoxin production in *A. flavus* by *L. plantarum* is limited. However, aflatoxin production in *A. parasiticus* can be inhibited by metabolites from LAB, and some components in the cell-free supernatant of *L. casei pseudoplantarum* 371, sensitive to proteolytic enzymes and heating, can inhibit aflatoxin synthesis in *A. parasiticus* [26]. Furthermore, metabolites from three LAB strains effectively reduced aflatoxin production in *A. parasiticus* [27].

It was reported that the drop in pH of the medium as a result of the growth of LAB strain *Streptococcus lactis* did not inhibit aflatoxin production by *A. flavus* [28]. In general, acidic pH favors the production of AFB_1_ by *Aspergillus* sp. [29,30]. Aflatoxin yields by *A. parasiticus* are increased in media with an initial pH of 4.2, compared with a pH close to neutrality [31]. In this study, the pH of the dual culture plates dropped from 6.0 to 3.5 after 3 days of incubation due to the production of organic acids; this drop in pH was not the main reason why aflatoxin production by *A. flavus* greatly decreased during the co-culture process. Hydrogen peroxide (H_2_O_2_) is produced by most LAB when oxygen is present [32]. Under dual culture with *A. flavus*, strain IAMU80070 did not produce H_2_O_2_ when grown in low-level media without oxygen. Therefore, inhibition of fungal growth and aflatoxin production did not appear to be associated with H_2_O_2_ production.

Sixteen genes in the aflatoxin synthesis pathway were downregulated, including *aflB* and *aflC*, that are involved in the formation of the aflatoxin starter unit. Downstream genes in this synthesis pathway may be downregulated due to the substrate inhibition effect.

In summary, we herein report for the first-time on the transcriptome-wide changes in *A. flavus* when co-cultured with *L. plantarum*.

## 4. Materials and Methods 

### 4.1. Chemicals, Media, Lactobacillus Plantarum and Aspergillus Strain

Aflatoxin B_1_, B_2_, G_1_, G_2_ (>99% purity) was purchased from Sigma-Aldrich (St. Louis, MI, USA). Stock standard solution at a concentration of 100 mg/kg was prepared with methanol (Thermo Fisher, Waltham, MA, USA). MRS medium (MRS; AOBOX, Beijing, China) was used for the isolation and cultivation of Lactobacillus strains [10]. A skim milk protective agent was used for the preservation of *Lactobacillus*.

Twenty-two strains of *L. plantarum* (Table 1) were donated by Professor Heping Zhang of the Inner Mongolia Agricultural University, all of which were isolated from dairy products, kimchi, sour porridge, and sour dough (Inner Mongolia, Sichuan, Xinjiang, Qinghai, Gansu and Tibet). For storage, *L. plantarum* strains were grown in MRS broth overnight and deep-frozen in skim milk protective agent at −80 °C until use. MRS was used for the reactivation of *L. plantarum*.

*A. flavus* strain ACCC 32656 was isolated from peanut-cropped soils (Huanggang, Hubei, China) and deposited in the Agricultural Culture Collection of China (ACCC). This strain, a highly toxigenic, S-type aflatoxin producer, was grown on potato dextrose agar (PDA; AOBOX, Beijing, China) slants at 28 °C for 7 days, and stored at 4 °C. Spores were collected from slants with sterile Tween-80 water (0.1% *v*/*v*) and adjusted to 10^7^ spores/mL with a haemocytometer [33].

### 4.2. Primary Screening of L. plantarum Strains Inhibiting A. flavus Activity

The inhibitory effects of *L. plantarum* strains against *A. flavus* were assayed as described previously [33] with minor modifications. *L. plantarum* strains were inoculated in two 2-cm lines on MRS agar plates, incubated at 37 °C for 24 h in anaerobic jars, and 10 mL of PDA (1% agar; AOBOX) containing 10^7^ spores/mL spores of *A. flavus* was added to the cultured plate. After 5 days of aerobic incubation at 28 °C, the zone of inhibition was measured. Plates not inoculated with *L. plantarum* strains served as controls. The antagonistic activity was assayed using a dual-culture method, then averaged, and assigned to one of three categories: +, slight inhibition with a discernible (<1 mm) clear zone from mycelial growth; ++, moderate inhibition with a 1-3 mm clear zone from mycelial growth; +++, high inhibition with a clear zone >3 mm from mycelial growth.

### 4.3. Effects of L. plantarum Strain IAMU80070 on A. flavus Growth

A suspension of *L. plantarum* strain IAMU80070 was prepared with MRS at concentrations of 1 × 10^6^, 3 × 10^6^, 1 × 10^7^ CFU/mL. A 1 mL aliquot of IAMU80070 suspension was added to 19 mL of MRS-melted solid agar and plated into Petri dishes, while the actual concentrations of strain IAMU80070 were 5 × 10^4^, 1.5 × 10^5^, 5 × 10^5^ CFU/mL. A 50 μL aliquot of fresh *A. flavus* conidia suspension (10^7^ spores/mL) was inoculated into the center of the plate and incubated at 28 °C for 5 days. A 1 mL aliquot of MRS medium was used instead of 1 mL of *L. plantarum* suspension as a control.

### 4.4. Effects of L. plantarum Strain IAMU80070 on Aflatoxin B1 Production by A. flavus

A 50 μL aliquot of fresh *A. flavus* conidia suspension (10^7^ spores/mL) was inoculated into the center of the 19 mL MRS-melted solid agar plate, which included a 1 mL AMU80070 suspension at a concentration of 1.5 × 10^5^ CFU/mL, and incubated at 28 °C for 8 days. The MRS medium was used instead of MRS-melted solid agar plate which included an IAMU80070 suspension as a control.

Aflatoxin B_1_ extraction and quantification were performed daily, according to a method reported previously [34]. Briefly, three agar plugs (1 × 1 cm) were taken from plates which were weighed and extracted with 1 mL chloroform. After centrifugation at 6000× *g* for 10 min, the supernatant was evaporated to dryness and redissolved in 200 μL of methanol: water (1:1, *v*/*v*). A high-performance liquid chromatography (HPLC) analysis was then performed according to our previous report [35].

The concentration of aflatoxin was defined as the content of aflatoxin per unit mass of hyphae, which was calculated according to the following formula:

$$\mathrm{the}\;\mathrm{concentration}\;\mathrm{of}\;\mathrm{aflatoxin}\;=\frac{C_{\mathrm{hplc}}\times 0.2}{m}$$

where C_hplc_ is the concentation measured by HPLC, and m is the mass of the three agar plugs.

### 4.5. Reduction of Aflatoxins Assay

AFB_1_, AFB_2_, AFG_1_, or AFG_2_ solutions (100 μg/mL; 0.01 mL respectively) were added to 1.96 mL suspensions of IAMU80070 obtain a final concentration of 0.5 μg/mL. The reduction test was performed for 3 days in a shaking incubator at 37 °C; sterile MRS containing AFB_1_,AFB_2_, AFG_1_, or AFG_2_ respectively at a concentration of 0.5 μg/mL was used as a control. Aflatoxin B1, B_2_, G_1_, and G_2_ extraction and quantification was performed and analyzed by HPLC according to a method reported previously [36].

### 4.6. Effects of Lactobacillus on the Ultrastructure of Aspergillus flavus

A 50 μL aliquot of fresh *A. flavus* conidia suspension (10^7^ spores/mL) was inoculated into the center of the MRS-melted solid agar plate which included an IAMU80070 suspension at a concentration of 1.5 × 10^5^ CFU/mL. A MRS medium was used instead of the MRS-melted solid agar plate which included IAMU80070 suspension as a control.

*L. plantarum*-treated and control plates were incubated at 28 °C for 5 days. The hyphae and spores of *A. flavus* were harvested for analysis by SEM. Samples were fixed, dehydrated, and coated according to the methods described in our previous study [24].

### 4.7. Production of Cell Wall Degrading Enzyme

The qualitative assay for chitinase production was performed according to the method described by Marten et al. [37]. IAMU80070 was inoculated as a single streak on the chitin-containing medium, the plates were incubated at 28 °C, and clearance halos around and beneath the growth indicating the enzymatic degradation were observed and measured after 5–10 days.

### 4.8. RNA Sequencing, Annotation and Analysis

A 50 μL aliquot of fresh *A. flavus* conidia suspension (10^7^ spores/mL) was inoculated into the center of the MRS-melted solid agar plate which included an IAMU80070 suspension at a concentration of 1.5 × 10^5^ CFU/mL. A MRS medium was used instead of a MRS-melted solid agar plate which included an IAMU80070 suspension as a control.

Based on fungal growth and aflatoxin production, the mycelia of *A. flavus* ACCC32656 cultured at 28 °C for 4 days were collected and the total RNA was isolated using a RNeasy Plant Mini Kit (Qiagen, Dusseldorf, Germany). Three biological replicates were made in the control (CK1, CK2, and CK3) and the *L. plantarum*-treated (T4, T5, and T7). The concentration and purity of the total RNA were determined using a NanoDrop 2000 spectrophotometer (Thermo Fisher Scientific, Waltham, MA, USA). The RNA quality was examined with an Agilent 2100 Bioanalyzer (Agilent Technologies, Santa Clara, CA, USA). The construction of an RNA-seq library was performed using a KAPA Stranded mRNA-Seq Kit for Illumina (KAPA Biosystems, Inc., Woburn, MA, USA) following the manufacturer’s instructions. Briefly, for total RNA, mRNA isolation, fragmentation and priming were performed using a KAPA mRNA capture system (Box1). Double-stranded cDNAs were synthesized using fragmented-primed mRNA, KAPA 1st Strand Synthesis Buffer, and KAPA Script, followed by KAPA 2nd Strand Marking Buffer and KAPA 2nd Strand Synthesis Enzyme Mix. Products were purified with 1.8× Agincourt AMPure XP beads (Beckman Coulter, Beverly, Brea, FL, USA) followed by 2^nd^ strand synthesis. After A-tailing, Illumina adapter oligonucleotides were ligated to cDNA fragments, and 1× SPRI clean-up was performed. Suitable cDNA fragments were selected as templates for PCR amplification using KAPA Library Amplification Primer Mix and KAPA HiFi HotStart Ready Mix. Products were purified using the AMPure XP bead system and quantified using a High Sensitivity Chip Bioanalyzer (Agilent Technologies, Santa Clara, CA, USA). Finally, RNA-seq libraries were sequenced using an Illumina HiSeq X Ten Microread Genetics Co. Ltd (Beijing, China). Raw data were processed with Fastp (0.12.6) using the recommended parameters (-w 8 -q 20 -l 50). Filtered reads were mapped to *Aspergillus flavus* NRRL3357 (https://www.ncbi.nlm.nih.gov/nuccore/NW_002477237.1) by HISAT2 [38] (2.0.1-beta), and bam files were processed with SAMtools [39] (Table 4). FeatureCounts was used to calculate gene expression. A list of differentially-expressed genes (DEGs) was identified using the R package “EdgeR”, and a *p*-value of 0.05 and |log2(fold-change)| > 2 were set as the thresholds for significant differential expression by default. GO and KEGG enrichment analyses of DEGs were implemented with KOBAS3.0. GO classification was performed with the R package ‘TopGO’.

### 4.9. Quantitative Real-Time RT-PCR (qRT-PCR) Analysis

To check the reproducibility and repeatability of gene expression data acquired by RNA-Seq, qRT-PCR analysis was performed as descried previously with independent samples collected at the same time as those used for RNA-Seq analysis [40,41]. The 18S RNA gene was used as an internal control to normalize the expression data. The relative expression of genes was calculated using the 2^−ΔΔCt^ method [42], and the standard deviation was calculated from three biological replicates. The gene specific primers used are listed in Supplemental Table S2.

### 4.10. Antifungal Activity in Bread

The antifungal assay was performed to check the activity of the biocontrol agent against the pathogen on bread according to the method described by Coda et al. [43]. A 10 μL inoculum of fresh *A. flavus* conidia suspension (10^7^ spores/mL) was added to a 4 mL IAMU80070 suspension (5 × 10^5^ CFU/mL). In this way, the ratio between *L. plantarum* and *A. flavus* was the same as on the plate in 4.3. Both suspensions were mixed and uniformly inoculated by the spray method on sliced breads, and incubated at 28 °C for 3 days. The mildew of the bread was observed. The control was maintained by using 4 mL MRS medium instead of an IAMU80070 suspension.

### 4.11. Antifungal Activity in Peanut Meal

An antifungal assay on peanut meal was conducted according to the method described by Ström et al. [44]. An *A. flavus* conidial suspension (10^7^ spores/mL) of volume 800 μL was added into 20 mL of IAMU80070 suspension (5 × 10^5^ CFU/mL). Both suspensions were evenly mixed and uniformly inoculated by spray method into 40g peanut meal and incubated at 28 °C. An antagonistic effect was observed after 3 days.

## Figures and Tables

**Table 1:** Characterization of *L. plantarum strains* for their potential to inhibit *A. flavus*.

Strain No.	Strain No.	Origin	Growth Inhibition ^a^
1	IMAU20063	Acid camel milk from Mongolian state	++
2	IMAU10570	Acid horse milk from Inner Mongolia Hulun Buir League	++
3	IMAU10704	Acid horse milk from Inner Mongolia Hulun Buir League	+
4	IMAU70164	Acid porridge from Hohhot City	+
5	IMAU40089	Acid yak milk from Qinghai Haibeizhou	+
6	IAMU80070	Kimchi from Huaiyuan Town, Chongzhou City, Sichuan Province	+++
7	IMAU80178	Kimchi from Pujiang County, Sichuan Province	+
8	IMAU60049	Yogurt from Suncheon, Tibet	++
9	IMAU80597	Qura from Xiahe County, Gansu Province	+
10	IMAU10725	Yogurt from Inner Mongolia Arukol Banner	+
11	IMAU10969	Yogurt from Inner Mongolia Bahrain Youqi Daban Town	+
12	IMAU10145	Goat milk from Bayannaoer City, Inner Mongolia	+
13	IMAU10124	Fermented cream from Bayannaoer City, Inner Mongolia	+
14	IMAU10278	Sour dough from Baotou City, Inner Mongolia	+
15	IMAU10386	Yogurt Inner Mongolia Hulunbeier League	+
16	IMAU10586	Sour horse milk from Hulunbeier League, Inner Mongolia	+
17	IMAU40001	Sour horse milk from Haixi, Qinghai	+
18	IMAU40091	Sour milk from Haibei, Qinghai	+
19	IMAU40100	Sour milk from Haibei, Qinghai	+
20	IMAU80441	Fresh milk from Aba, Sichuan	+
21	IMAU60026	Sour milk from Shigatse Prefecture, Tibet	+
22	IMAU30001	Sour horse milk from Xinjiang Yili Prefecture	+

^a^ Growth inhibition of all strains was assayed using a dual-culture method in MRS agar plates, then averaged, and assigned to one of three categories: +, slight inhibition with a discernible (<1 mm) clear zone from mycelial growth; ++, moderate inhibition with a 1-3 mm clear zone from mycelial growth; and +++, high inhibition with a clear zone >3 mm from mycelial growth.

**Table 2:** The reduction of aflatoxins by *L. plantarum* IAMU80070.

No.	Strain No.	AFB_1_ Reduction	AFB_2_ Reduction	AFG_1_ Reduction	AFG_2_ Reduction
1	IAMU80070	7.9% ± 2.9%	5.2% ± 0.5%	2.3% ± 1.5%	6.4% ± 0.1%

**Table 3:** Reads and reference genome comparison.

Sample	Total Reads	Mapped Reads	Mapping Rate (%)
CK1	43,060,056	37,785,199	87.75
CK2	39,269,612	34,231,320	87.17
CK3	41,770,838	35,939,629	86.04
T4	39,589,518	33,710,475	85.15
T5	38,192,168	32,505,354	85.11
T7	40,226,730	34,502,466	85.77

**Table 4:** KEGG pathways of differentially expressed genes with GO terms enriched.

Term	ID	Gene Number	Rich Factor	*p*-Value	Corrected *p*-Value	Note
Aflatoxin biosynthesis	afv00254	7	0.50	2.3 × 10^−10^	6.5 × 10^−9^	Down regulated
Pentose phosphate pathway	afv00030	3	0.10	2.7 × 10^−3^	3.9 × 10^−2^	Down regulated
Pyruvate metabolism	afv00620	3	0.08	5.3 × 10^−3^	4.0 × 10^−2^	Down regulated
Carbon metabolism	afv01200	5	0.04	5.5 × 10^−3^	4.0 × 10^−2^	Down regulated

## Supplementary Materials

Supplementary materials can be accessed at: https://www.mdpi.com/2072-6651/11/11/636/s1, Table S1: The Differential expressed genes among RNA-seq groups; Table S2: The gene specific primers of qRT-PCR.
