# Supplementary material for: Morphological and Transcriptomic Analysis of the Inhibitory Effects of Lactobacillus plantarum on Aspergillus flavus Growth and Aflatoxin Production
Source: Toxins (Basel). 2019 Nov 1;11(11):636. doi: 10.3390/toxins11110636 (PMC6891564; doi:10.3390/toxins11110636)
Supplement: Supplementary file 1 [file toxins-11-00636-s001.zip › toxins-630448 supplementary revised/toxins-630448 supplementary table S2.pdf]

Supplementary Materials: Morphological and Transcriptomic Analysis of the Inhibitory Effects of *Lactobacillus plantarum* on *Aspergillus flavus* Growth and Aflatoxin Production

Yueju Zhao, Chenxi Zhang, Yawa Minnie Elodie Folly, Jinghua Chang, Yan Wang, Lu Zhou and Heping Zhang and Yang Liu

Table S2. The gene specific primers of qRT-PCR.

Gene name [a]	Primer Name	Primer sequence (5′-3′)	Product size (bp)
18S RNA	18S RNA-F	TGACAATGGGCTCAGAGGATG	95 bp
	18S RNA-R	TTTCGCTAAGTCTCTCGCCAG	
aflD	aflD-F	ATGCTCCCGTCCTACTGTTT	106 bp
	aflD-R	ATGTTGGTGATGGTGCTGAT	
aflG	aflG-F	TAACTTCCATCGTCCCGAAGAG	142bp
	aflG-R	CATTTCCACGTAGGCTAGCTGTC	
aflI	aflI-F	CACAACCAACGGGCTACAGAC	218 bp
	aflI-R	TATTTTCTCCCTCAAACGAGCC	
aflK	aflK-F	GCTGGGCATTCCAGTACGAT	114 bp
	aflK-R	CCCATCAACTGACTGTGGCT	
aflR	aflR-F	AGTGAACTGCACCGAGTCCAG	116 bp
	aflR-R	AGTAGTGAGAAAGGGGACGCTG	
catA	catA-F	CGACGATGTTTGACGCTACCT	231 bp
	catA-R	CAGCCGTGACAACACCATACC	
7910898	7910898-F	AAGCAATCGGACTGTCCAACC	171 bp
	7910898-R	GCCCTTTTCCCACTCATCGT	
7912413	7912413-F	GGCCTGTGGATACCAAACCA	168 bp
	7912413-R	TAGTAGGGGTTCTGCTTGACGC	

[a] 7910898 (GeneID) encodes a class V chitinase Chi100; 7912413 (GeneID) encodes a putative extracellular endoglucanase/cellulase.
